# *N* ^1^,*N* ^2^-Di­methyl­ethane-1,2-diaminium dichloride

**DOI:** 10.1107/S1600536814001627

**Published:** 2014-01-29

**Authors:** Joseph S. Merola

**Affiliations:** a Department of Chemistry 0212, Virginia Tech, Blacksburg, VA 24061, USA

## Abstract

The cation of the title salt, C_4_H_14_N_2_^2+^·2Cl^−^, is located on a crystallographic inversion center and is bis­ected by a mirror plane, with one quarter of the C_4_H_14_N_2_^2+^·2Cl^−^ formula unit being crystallographically unique. he chloride ions also sit on a mirror plane. The conformation of the cation is a regular straight-chain conformation with all non-H atoms in *anti* positions. In the crystal, hydrogen bonding between N—H groups and chloride anions yields a zigzag ladder-type structure along [010].

## Related literature   

For the crystal structure of *N* ^1^,*N* ^2^-di­methyl­ethane-1,2-diam­in­ium di­thio­cyanate (CCDC: 662389), see: Wolstenholme *et al.* (2008 ▶), of ethane-1,2-diaminium]chloride (CCDC: 790989), see: Liu *et al.* (2010 ▶), of *N* ^1^,*N* ^1^,*N* ^2^,*N* ^2^-tetra­methyl­ethane-1,2-diaminium dichloride, see: Schneider & Schier (2004 ▶; CCDC: 247442) and Kabak *et al.*, 2000 ▶; CCDC: 142944) and of *N* ^1^,*N* ^1^,*N* ^2^-tri­methyl­ethylenedi­ammonium dichloride, see: Errington *et al.* (2001 ▶). The most recent description of the Cambridge Crystallographic Database can be found in Groom & Allen (2014 ▶).
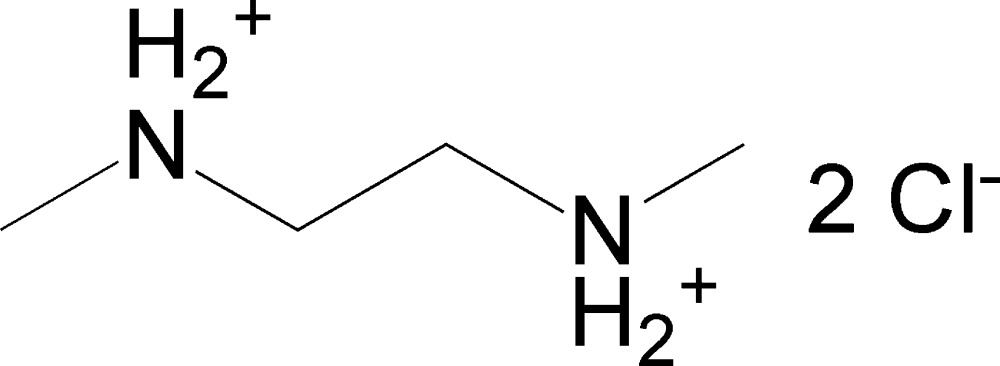


## Experimental   

### 

#### Crystal data  

C_4_H_14_N_2_^2+^·2Cl^−^
*M* *_r_* = 161.07
Monoclinic, 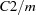

*a* = 18.108 (2) Å
*b* = 5.104 (1) Å
*c* = 5.080 (1) Å
β = 105.09 (3)°
*V* = 453.32 (14) Å^3^
*Z* = 2
Mo *K*α radiation
μ = 0.64 mm^−1^
*T* = 293 K
0.4 × 0.2 × 0.2 mm

#### Data collection  

Siemens P4 diffractometer
590 measured reflections
443 independent reflections
398 reflections with *I* > 2σ(*I*)
*R*_int_ = 0.022
3 standard reflections every 300 reflections intensity decay: none

#### Refinement  

*R*[*F* ^2^ > 2σ(*F* ^2^)] = 0.026
*wR*(*F* ^2^) = 0.070
*S* = 1.14
443 reflections
41 parameters
H atoms treated by a mixture of independent and constrained refinement
Δρ_max_ = 0.24 e Å^−3^
Δρ_min_ = −0.15 e Å^−3^

### 

Data collection: *XSCANS* (Siemens, 1996 ▶); cell refinement: *XSCANS*; data reduction: *XSCANS*; program(s) used to solve structure: *SHELXS97* (Sheldrick, 2008 ▶; program(s) used to refine structure: *SHELXL97* (Sheldrick, 2008 ▶); molecular graphics: *OLEX2* (Dolomanov *et al.*, 2009 ▶); software used to prepare material for publication: *OLEX2*.

## Supplementary Material

Crystal structure: contains datablock(s) I. DOI: 10.1107/S1600536814001627/zl2574sup1.cif

Structure factors: contains datablock(s) I. DOI: 10.1107/S1600536814001627/zl2574Isup2.hkl

Supporting information file. DOI: 10.1107/S1600536814001627/zl2574Isup3.mol

Supporting information file. DOI: 10.1107/S1600536814001627/zl2574Isup4.cml

CCDC reference:

Additional supporting information:  crystallographic information; 3D view; checkCIF report

## Figures and Tables

**Table 1:** Hydrogen-bond geometry (Å, °)

*D*—H⋯*A*	*D*—H	H⋯*A*	*D*⋯*A*	*D*—H⋯*A*
N1—H1⋯Cl1^i^	0.94 (2)	2.13 (2)	3.0741 (13)	176.2 (17)

Symmetry code: (i) 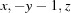.

**Fig. 1.**
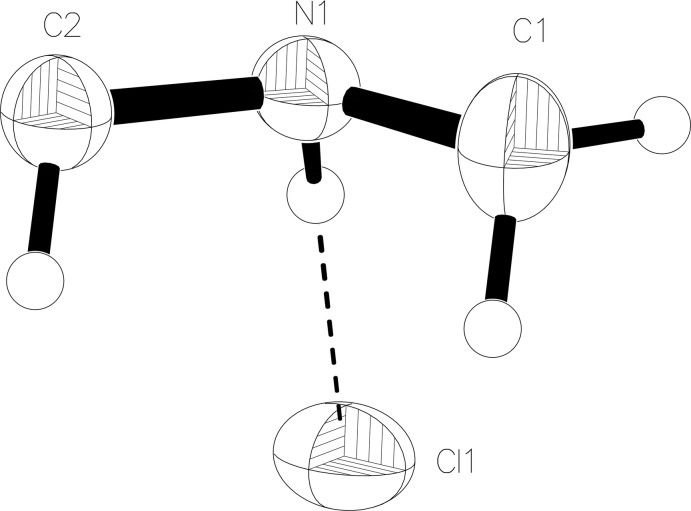
Thermal ellipsoid drawing of the asymmetric unit of the title compound. Ellipsoids are drawn at the 50% probability level.

**Fig. 2.**
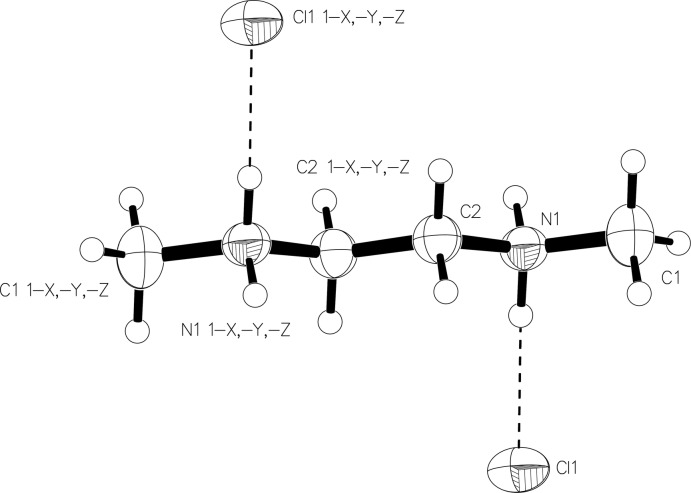
Thermal ellipsoid of the title molecule. Ellipsoids are drawn at the 50% probability level. Symmetry-generated atoms are labeled with the symmetry operator that generated them.

**Fig. 3.**
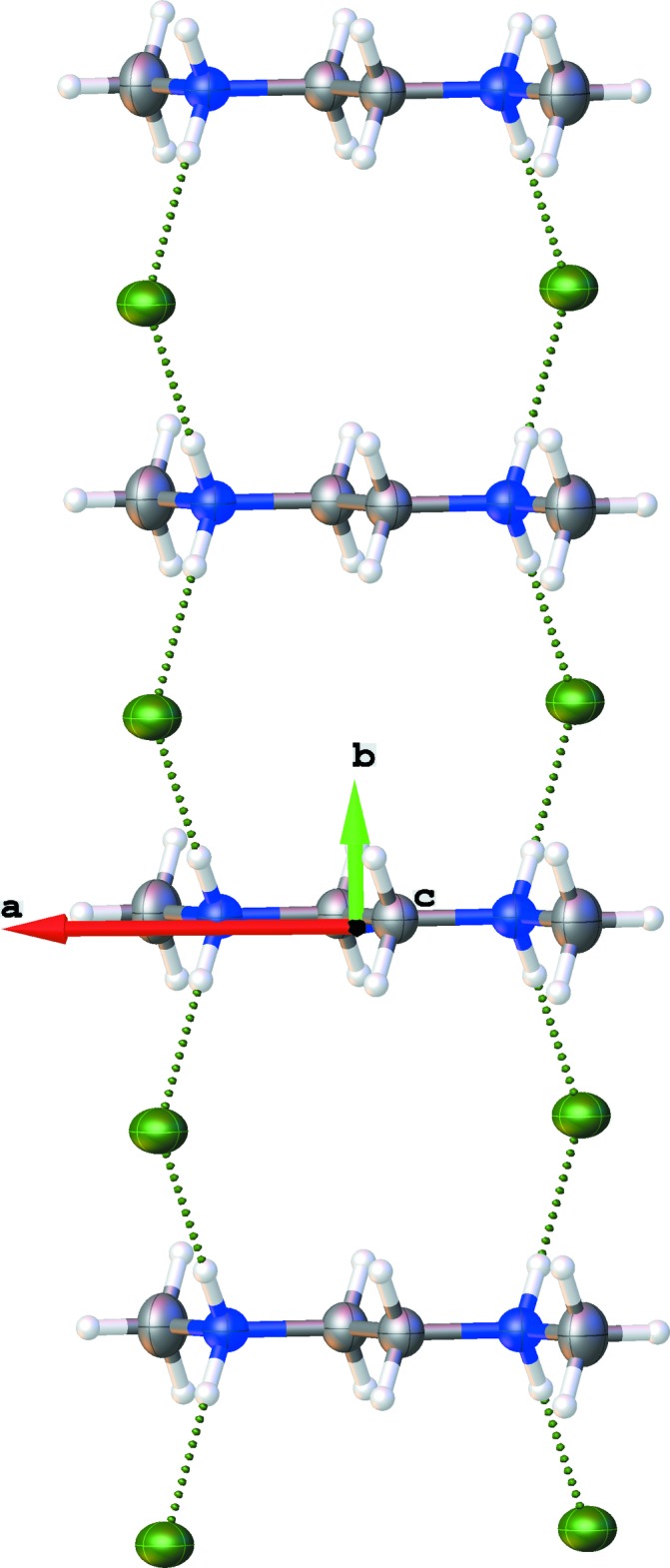
A view of the hydrogen-bonding in the title compound showing the zigzag ladder-like H-bonding motif. View is down the c-axis.

**Fig. 4.**
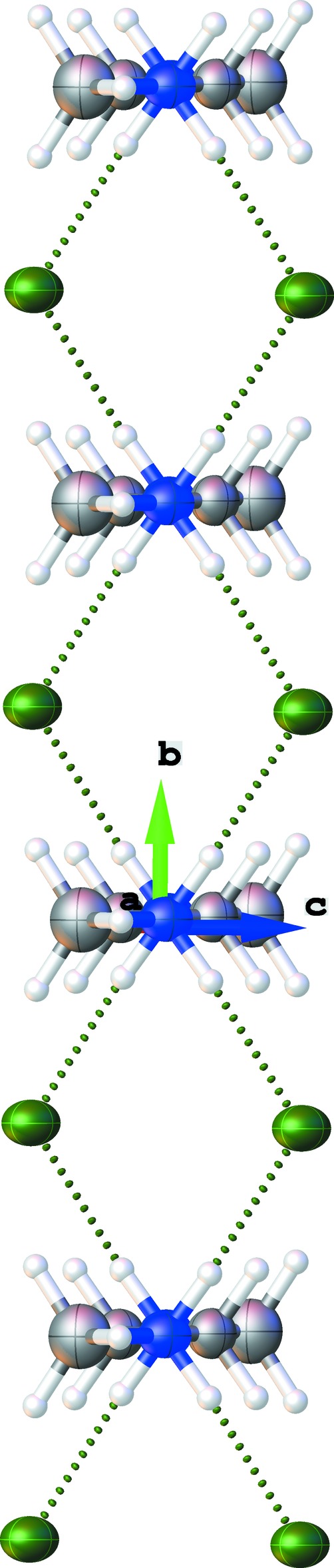
A view of the hydrogen-bonding in the title compound showing a side view of the ladder-like hydrogen-bonding motif. View is down the a-axis.

## supplementary crystallographic information

### 1. Comment

Ethane-1,2-diaminium cations and *N*-alkyl substituted variants play a large role in structural chemistry as counter-ions for quite a number of anions and anionic complexes. A search of the Cambridge Crystallographic Database shows hundreds of structures in which some form of ethane-1,2-diaminium salt serves as the cation (Groom & Allen, 2014). There are many fewer examples of simple halide salts of these dications and there are no simple halide salts of the [*N*^1^,*N*^2^-dimethylethane-1,2-diaminium] moiety. This report discusses the structure of [*N*^1^,*N*^2^-dimethylethane-1,2-diaminium]chloride and the hydrogen-bonding motif set up in the lattice. While the *N*^1^,*N*^2^-ethylenediaminium cation has been structurally characterized as the thiocyanate salt (Wolstenholme *et al.*, 2008) as well as with a number of metal complex anions, the structure of the chloride salt has not been determined previously.

The title compound crystallizes in the C2/m space group and the molecule is located on a crystallographic inversion center and is bisected by a mirror plane, with one quarter of a molecule of [C_4_H_14_N_2_]^2+^ 2Cl^-^ being crystallographically unique. Figure 1 shows the thermal ellipsoid plot of the asymmetric unit and Figure 2 shows the thermal ellipsoid plot of the entire molecule with atoms labeled with name and symmetry operation that generates them. The diaminium cation adopts a classical linear geometry with all heavy atoms lying in a plane and all bond angles being ± one degree from the ideal tetrahedrality.

The presence of two hydrogen atoms on each nitrogen atom leads to a ladder hydrogen-bonding motif with each N—H bonding to a chloride anion. Figure 3 shows a view down the *c*-axis showing the ladder arrangement while figure 4 shows the same arrangement down the *a*-axis.

Other ethanediaminium chlorides have been structurally characterized, but all have very different hydrogen bonding motifs. Obviously, hexamethylethylenediaminium chloride has no N—H groups and therefore, no H-bonding, so the lack of a chloride ion comparison for that dication is not important. The unsubstituted ethylenediaminium chloride, having 3 N—H hydrogen bonds to the chloride ion has a more complex, three-dimensional H-bonding network (Liu *et al.*, 2010). The [*N*^1^,*N*^1^,*N*^2^,*N*^2^-tetramethylethane-1,2-diaminium]chloride structure, with only one N—H per nitrogen atom, shows clear hydrogen-bonding, but there is no extended lattice structure, just isolated N—H···Cl bonds (Schneider & Schier, 2004) and (Kabak *et al.*, 2000). The trimethyl compound, [*N*^1^,*N*^1^,*N*^2^-trimethyldiaminium chloride has a complicated structure with two different conformations of the dication in the asymmetric unit, each hydrogen-bonding to the chloride ions in different ways and yielding an iregular motif (Errington *et al.*, 2001).

### 2. Experimental

The crystal used in this experiment was obtained from a reaction between [Ir(COD)Cl]_2_ (COD = 1,5-cyclooctadiene)(0.100 g) and *N*^1^,*N*^2^-ethane-1,2-diamine (0.200 g) in dichloromethane solution. After stirring at room temperature overnight, the solvent was removed under reduced pressure to yield 0.147 g of a white powder. Some of the powder was dissolved in dichloromethane and allowed to evaporate slowly in a vial with the serum cap punctured with a needle. Upon evaporation of all of the solvent, most of the solid was a powder with only a few nicely shaped crystals in the mixture. A crystal was chosen and epoxied onto a thin quartz fiber and placed in a goniometer. Apparently, on sitting, some amount of decomposition occurred to yield the free diamine as well as the generation of hydrogen chloride resulting in the formation of the title compound.

### 3. Refinement

All hydrogen atoms in the structure were located by difference map and all parameters not fixed by location on a symmetry element (mirror plane) were refined. N—H bonds are 0.94 (2) Å (Table 1) and are identical by symmetry (mirror plane and inversion center).

### Crystal data

**Table:**

C_4_H_14_N_2_^2+^·2Cl^−^	*F*(000) = 172
*M**_r_* = 161.07	*D*_x_ = 1.180 Mg m^−^^3^
Monoclinic, *C*2/*m*	Mo *K*α radiation, λ = 0.71073 Å
*a* = 18.108 (2) Å	Cell parameters from 35 reflections
*b* = 5.104 (1) Å	θ = 2.2–20°
*c* = 5.080 (1) Å	µ = 0.64 mm^−^^1^
β = 105.09 (3)°	*T* = 293 K
*V* = 453.32 (14) Å^3^	Irregular, clear colourless
*Z* = 2	0.4 × 0.2 × 0.2 mm

### Data collection

**Table:**

Siemens P4 diffractometer	*R*_int_ = 0.022
Radiation source: fine-focus sealed tube	θ_max_ = 25.0°, θ_min_ = 2.3°
Graphite monochromator	*h* = −1→21
ω scans	*k* = −1→6
590 measured reflections	*l* = −6→5
443 independent reflections	3 standard reflections every 300 reflections
398 reflections with *I* > 2σ(*I*)	intensity decay: 0.0(1)

### Refinement

**Table:**

Refinement on *F*^2^	Secondary atom site location: difference Fourier map
Least-squares matrix: full	Hydrogen site location: difference Fourier map
*R*[*F*^2^ > 2σ(*F*^2^)] = 0.026	H atoms treated by a mixture of independent and constrained refinement
*wR*(*F*^2^) = 0.070	*w* = 1/[σ^2^(*F*_o_^2^) + (0.0266*P*)^2^ + 0.2719*P*] where *P* = (*F*_o_^2^ + 2*F*_c_^2^)/3
*S* = 1.14	(Δ/σ)_max_ < 0.001
443 reflections	Δρ_max_ = 0.24 e Å^−^^3^
41 parameters	Δρ_min_ = −0.15 e Å^−^^3^
0 restraints	Extinction correction: *SHELXL97* (Sheldrick, 2008), Fc^*^=kFc[1+0.001xFc^2^λ^3^/sin(2θ)]^-1/4^
Primary atom site location: iterative	Extinction coefficient: 0.041 (6)

### Special details

**Table:**

Geometry. All e.s.d.'s (except the e.s.d. in the dihedral angle between two l.s. planes) are estimated using the full covariance matrix. The cell e.s.d.'s are taken into account individually in the estimation of e.s.d.'s in distances, angles and torsion angles; correlations between e.s.d.'s in cell parameters are only used when they are defined by crystal symmetry. An approximate (isotropic) treatment of cell e.s.d.'s is used for estimating e.s.d.'s involving l.s. planes.
Refinement. Refinement of *F*^2^ against ALL reflections. The weighted *R*-factor *wR* and goodness of fit *S* are based on *F*^2^, conventional *R*-factors *R* are based on *F*, with *F* set to zero for negative *F*^2^. The threshold expression of *F*^2^ > σ(*F*^2^) is used only for calculating *R*-factors(gt) *etc*. and is not relevant to the choice of reflections for refinement. *R*-factors based on *F*^2^ are statistically about twice as large as those based on *F*, and *R*- factors based on ALL data will be even larger.

### Fractional atomic coordinates and isotropic or equivalent isotropic displacement parameters (Å^2^)

**Table:**

	*x*	*y*	*z*	*U*_iso_*/*U*_eq_	
N1	0.60591 (11)	0.0000	0.0428 (4)	0.0381 (5)	
H1	0.6075 (10)	−0.149 (4)	−0.066 (4)	0.055 (5)*	
C1	0.67523 (16)	0.0000	0.2769 (7)	0.0564 (8)	
H1A	0.7175 (18)	0.0000	0.198 (6)	0.067 (9)*	
H1B	0.6737 (13)	−0.167 (5)	0.385 (4)	0.078 (7)*	
C2	0.53327 (13)	0.0000	0.1259 (5)	0.0393 (6)	
H2	0.5317 (9)	−0.153 (4)	0.234 (3)	0.045 (5)*	
Cl1	0.61356 (4)	−0.5000	−0.28640 (13)	0.0501 (3)	

### Atomic displacement parameters (Å^2^)

**Table:**

	*U*^11^	*U*^22^	*U*^33^	*U*^12^	*U*^13^	*U*^23^
N1	0.0376 (11)	0.0323 (11)	0.0462 (12)	0.000	0.0143 (9)	0.000
C1	0.0378 (15)	0.067 (2)	0.0617 (17)	0.000	0.0088 (13)	0.000
C2	0.0383 (13)	0.0409 (14)	0.0402 (13)	0.000	0.0130 (10)	0.000
Cl1	0.0634 (5)	0.0343 (4)	0.0576 (4)	0.000	0.0246 (3)	0.000

### Geometric parameters (Å, º)

**Table:**

N1—H1	0.94 (2)	C1—H1B	1.02 (2)
N1—C1	1.488 (3)	C2—C2^i^	1.511 (5)
N1—C2	1.482 (3)	C2—H2	0.960 (18)
C1—H1A	0.95 (3)	Cl1—Cl1^ii^	0.0000 (13)
			
C1—N1—H1	108.6 (11)	H1A—C1—H1B	111.5 (15)
C2—N1—H1	109.3 (11)	N1—C2—C2^i^	109.3 (2)
C2—N1—C1	113.5 (2)	N1—C2—H2	108.8 (10)
N1—C1—H1A	105.4 (18)	C2^i^—C2—H2	110.4 (10)
N1—C1—H1B	107.2 (13)		
			
C1—N1—C2—C2^i^	180.0		

Symmetry codes: (i) −*x*+1, −*y*, −*z*; (ii) *x*, −*y*−1, *z*.

### Hydrogen-bond geometry (Å, º)

**Table:**

*D*—H···*A*	*D*—H	H···*A*	*D*···*A*	*D*—H···*A*
N1—H1···Cl1^ii^	0.94 (2)	2.13 (2)	3.0741 (13)	176.2 (17)

Symmetry code: (ii) *x*, −*y*−1, *z*.
